# And Yet It Moves: Clinical Outcomes and Motion Management in Stereotactic Body Radiation Therapy (SBRT) of Centrally Located Non-Small Cell Lung Cancer (NSCLC): Shedding Light on the Internal Organ at Risk Volume (IRV) Concept

**DOI:** 10.3390/cancers16010231

**Published:** 2024-01-04

**Authors:** Felix-Nikolai Oschinka Jegor Habermann, Daniela Schmitt, Thomas Failing, David Alexander Ziegler, Jann Fischer, Laura Anna Fischer, Manuel Guhlich, Stephanie Bendrich, Olga Knaus, Tobias Raphael Overbeck, Hannes Treiber, Alexander von Hammerstein-Equord, Raphael Koch, Rami El Shafie, Stefan Rieken, Martin Leu, Leif Hendrik Dröge

**Affiliations:** 1 Department of Radiotherapy and Radiation Oncology, University Medical Center Göttingen, Robert-Koch-Str. 40, 37075 Göttingen, Germany; felix.habermann@med.uni-goettingen.de (F.-N.O.J.H.); daniela.schmitt@med.uni-goettingen.de (D.S.); alexander.ziegler@med.uni-goettingen.de (D.A.Z.); jann.fischer@med.uni-goettingen.de (J.F.); laura-anna.fischer@med.uni-goettingen.de (L.A.F.); manuel.guhlich@med.uni-goettingen.de (M.G.); stephanie.bendrich@med.uni-goettingen.de (S.B.); rami.elshafie@med.uni-goettingen.de (R.E.S.); stefan.rieken@med.uni-goettingen.de (S.R.); martin.leu@med.uni-goettingen.de (M.L.); 2 Göttingen Comprehensive Cancer Center (G-CCC), University Medical Center Göttingen, Von-Bar-Str. 2/4, 37075 Göttingen, Germany; tobias.overbeck@med.uni-goettingen.de (T.R.O.); hannes.treiber@med.uni-goettingen.de (H.T.); alexander.hammerstein@med.uni-goettingen.de (A.v.H.-E.); raphael.koch@med.uni-goettingen.de (R.K.); 3 Institute of Medical Physics and Radiation Protection (IMPS), University of Applied Sciences, Wiesenstr. 14, 35390 Gießen, Germany; 4 Department of Hematology and Medical Oncology, University Medical Center Göttingen, Robert-Koch-Str. 40, 37075 Göttingen, Germany; 5 Department of Cardio-Thoracic and Vascular Surgery, University Medical Center Göttingen, Robert-Koch-Str. 40, 37075 Göttingen, Germany

**Keywords:** NSCLC, SBRT, central tumors, clinical outcomes, clinical characteristics, motion management, planning organ at risk volume, PRV, internal organ at risk volume, IRV

## Abstract

**Simple Summary:**

We studied clinical aspects in central vs. peripheral tumors (*n* = 78 patients) and applied the internal organ at risk volume (IRV) concept (*n* = 35 patients) in stereotactic body radiation therapy (SBRT) for centrally located non-small cell lung cancer (NSCLC)**.** We found lower biologically effective doses, larger planning target volume sizes, higher lung doses, and worse locoregional control for central tumors when compared with peripheral tumors. We here provide evidence that organ motion/volume changes could be more pronounced in males and tall patients, and less pronounced in cases of higher body mass index. Applying the IRV concept (retrospectively, without new optimization), the normal tissue complication probabilities increased >10% for the bronchial tree in three patients. This study emphasizes the need to optimize methods to balance dose escalation with toxicities in central tumors. Since recent studies have made efforts to further subclassify central tumors to refine treatment, the IRV concept should be considered for optimal risk assessment.

**Abstract:**

The internal organ at risk volume (IRV) concept might improve toxicity profiles in stereotactic body radiation therapy (SBRT) for non-small cell lung cancer (NSCLC). We studied (1) clinical aspects in central vs. peripheral tumors, (2) the IRV concept in central tumors, (3) organ motion, and (4) associated normal tissue complication probabilities (NTCPs). We analyzed patients who received SBRT for NSCLC (clinical aspects, *n* = 78; motion management, *n* = 35). We found lower biologically effective doses, larger planning target volume sizes, higher lung doses, and worse locoregional control for central vs. peripheral tumors. Organ motion was greater in males and tall patients (bronchial tree), whereas volume changes were lower in patients with a high body mass index (BMI) (esophagus). Applying the IRV concept (retrospectively, without new optimization), we found an absolute increase of >10% in NTCPs for the bronchial tree in three patients. This study emphasizes the need to optimize methods to balance dose escalation with toxicities in central tumors. There is evidence that organ motion/volume changes could be more pronounced in males and tall patients, and less pronounced in patients with higher BMI. Since recent studies have made efforts to further subclassify central tumors to refine treatment, the IRV concept should be considered for optimal risk assessment.

## 1. Introduction

In patients with early-stage non-small cell lung cancer (NSCLC) who are not suitable for surgery or refuse surgery, stereotactic body radiation therapy (SBRT) is an effective treatment option [1]. In stage I patients, SBRT achieves 2-year local control rates of >90% [2].

For SBRT, it has been demonstrated that higher biologically effective doses (≥100–125 Gy, alpha/beta ratio of 10 [BED10]) are crucial to achieve these excellent control rates [3,4]. At the same time, the tumors are located centrally in about 44% [5] of cases. Patients with tumors in central or ultra-central locations are at an increased risk of complications, e.g., bronchial stenosis or esophageal perforation [6,7,8,9]. Timmermann et al. reported that 46% of patients with central tumors experienced severe toxicities [10].

Thus, the maintenance of high-quality standards and continuous development of SBRT planning and delivery are required for safe and effective treatment [4,11]. Here, the management of structure motion within the breathing cycle plays a crucial role [11]. A four-dimensional CT scan (4D-CT) with the internal target volume (ITV) concept is widely applied to account for tumor motion [11,12]. The planning organ at risk volume concept (PRV) might help to reduce the probability of organs at risk (OARs) overdosage [13,14]. The concept is less well-studied and rarely used in clinical practice [14,15]. Additionally, intrafractional structure movement is not taken into account [15]. Here, the internal organ at risk volume (IRV) concept might be advantageous [15,16]. Nardone et al. found unacceptable treatment plans in 42% of the cases with central tumors when applying the IRV concept in SBRT for NSCLC [15].

In summary, SBRT in patients with centrally located NSCLC is a substantial challenge for the treatment team [17]. In this study, we compared clinical characteristics and outcomes in peripherally located vs. centrally located tumors in patients who received SBRT in the local radiotherapy department. Additionally, we analyzed the deviation of the geometric centers (OAR vs. IRV) and the volume differences (OAR vs. IRV) in serial OARs (bronchial tree, trachea, esophagus, and spinal canal) throughout the breathing cycle. We tested for an influence of patient-related characteristics on organ motion. Additionally, we evaluated whether the application of the IRV concept (retrospectively, without new optimization) leads to a relevant increase in normal tissue complication probabilities (NTCPs).

## 2. Patients and Methods

### 2.1. Study Design

**Figure 1:**
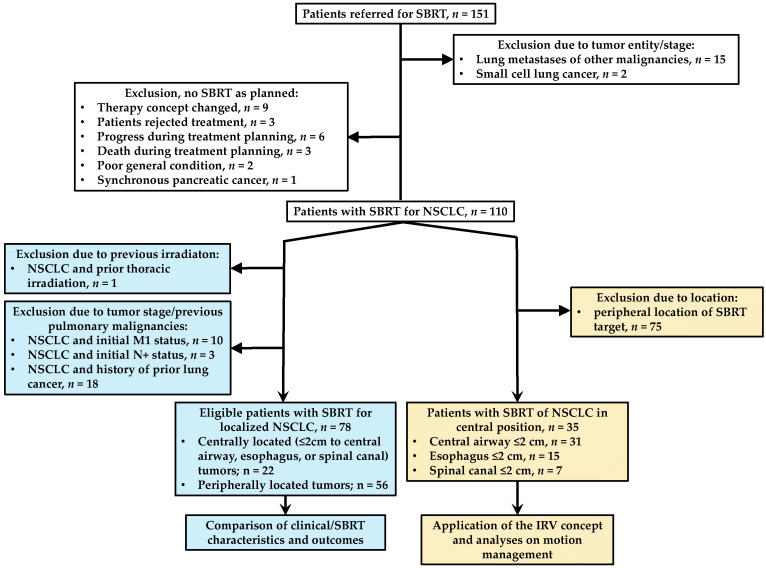
The flow chart illustrates the patient selection for the comparison of clinical characteristics in centrally vs. peripherally located tumors (left side, blue background) and the studies on motion management for OARs (right side, orange background). SBRT: stereotactic body radiation therapy. OARs: organs at risk. IRV: internal organ at risk volume.

We first identified all the patients in the medical records who were referred to the radiation oncology department for SBRT to the lungs or the mediastinal structures. A total of 151 patients were documented. We previously performed a study as part of the underlying project on patterns of pretreatment diagnostic assessment with a special focus on the COVID-19 pandemic [18]. Here, we present a study that differed in patient selection (Figure 1), methods, and outcome parameters. For the comparison of clinical characteristics and outcomes in centrally vs. peripherally located tumors, only patients with localized NSCLC (*n* = 78 patients) were included. For the analysis of motion management for OARs, we included all the patients with SBRT for NSCLC in a central tumor position (*n* = 35). Chang et al. recommended choosing a distance of 2 cm from any critical mediastinal structure as the cut-off for a central position [19]. In this study, we focused on tumors with a distance of ≤2 cm to the serial organs, bronchial tree and trachea (together, central airway), esophagus, and spinal canal. Please see Figure 1 for further details on patient selection. The study was approved by the local ethics committee of the University Medical Center Göttingen (application no. 3/10/20).

### 2.2. Radiotherapy, Planning and Delivery

Please see Habermann et al. in 2022 for a previous description of SBRT [18]. A 4D-CT with a respiration belt and the patient in a supine position was acquired for treatment planning. For patient positioning, customized devices were used. The ITV concept was used for target volume delineation [11,12]. The gross tumor volume (GTV) was delineated in all of the 10 breathing phases. The ITV was generated by including the GTVs in all the phases. The planning target volume (PTV) was created with individual margins, which were left at the discretion of the treating radiation oncologist (between 3 mm and 10 mm). For treatment planning, the software, Eclipse (Varian Medical Systems, Palo Alto, CA, USA), was used. We applied the Eclipse versions 10.0 (12/2012–09/2013), 11.1 (10/2013–09/2014), 13.5 (10/2014–05/2020), and 15.6 (from 06/2020). Radiotherapy was applied with Varian Clinac 2300 CD linear accelerators (Varian Medical Systems, Palo Alto, CA, USA). Daily cone beam CT was used for image guidance. Please see Section 2.2.1 and Section 2.2.2 for further details on the patient cohorts.

#### 2.2.1. Patient Cohort with Analysis of Clinical/SBRT Characteristics and Outcomes

We used the Philips Gemini TF TOF 16 (*n* = 22 patients), Philips Ingenuity Flex (*n* = 3 patients), and Philips Brilliance Big Bore (*n* = 53 patients) for the acquisition of the CT scans (each, Philips Medical Systems, Fitchburg, WI, USA). The slice thicknesses were 2 mm (*n* = 4 patients) and 3 mm (*n* = 72 patients). In 2 patients, a larger slice thickness of 5 mm was chosen on an individual basis in the clinical routine. We decided to include these 2 patients in the analysis of clinical outcomes since technical aspects were not the main endpoints in this part of the project. We used the algorithms Acuros (*n* = 70 patients) and AAA (*n* = 18 patients). In 61 patients, the prescription isodose was 80%. In 17 patients, radiotherapy was prescribed homogeneously.

#### 2.2.2. Patient Cohort with Analyses on Motion Management

The CT scanners were the Philips Gemini TF TOF 16 (*n* = 11 patients), Philips Ingenuity Flex (*n* = 2 patients), and Philips Brilliance Big Bore (*n* = 22 patients) (each, Philips Medical Systems, Fitchburg, WI, USA). The slice thickness was 3 mm in all the patients (*n* = 35). We used the algorithm Acuros in all the patients (*n* = 35). The prescription isodose was 80% in 26 patients. In 9 patients, radiotherapy was prescribed homogeneously.

### 2.3. Endpoints and Statistical Methods

#### 2.3.1. Patient Cohort with Analysis of Clinical/SBRT Characteristics and Outcomes

Please see Habermann et al. in 2022 for a previous description of statistical approaches in the underlying project [18]. We compared characteristics and outcomes between patients with centrally located and peripherally located tumors. When comparing baseline and SBRT characteristics, we used Pearson’s Chi-squared test and the Mann–Whitney U test (SPSS v. 27, IBM, Armonk, NY, USA). In survival analyses, the endpoints were overall survival (OS, event: patient death due to any cause), progression-free survival (PFS, event: locoregional or distant progression and patient death), local progression-free survival (LPFS, events: local progression and patient death due to any cause), and locoregional control (LRC, events: local or regional relapse). The survival times were calculated from the first day of SBRT. We used Cox regression analysis (SPSS v. 27, IBM, Armonk, NY, USA). Additionally, the Kaplan–Meier curves with log-rank statistics were generated using the plugin, KMWin v 1.53 [20]. *p*-values < 0.05 were considered statistically significant.

#### 2.3.2. Patient Cohort with Analyses on Motion Management

In the subset of 35 patients with SBRT of NSCLC in a central position, the IRV concept was applied (retrospectively, without new optimization). The 4D-CTs were used and the OARs (bronchial tree, trachea, esophagus, and spinal canal) were contoured on each of the 10 respiratory phases and the average intensity projection (AIP) CT scan. The OARs were delineated in accordance with the Radiation Therapy Oncology Group guidelines [21]. The bronchial tree was defined as including the distal 2 cm of the trachea, the carina, the main bronchi, the right and left upper lobe bronchi, the bronchus intermedius, the right middle lobe bronchus, the lingular bronchus, and the right and left lower lobe bronchi [21]. The trachea was delineated from the lower edge of the larynx to the bronchial tree. The esophagus was contoured from just below the cricoid to the gastroesophageal junction [21]. The spinal canal was defined based on the surrounding bones [21]. We used the thoracic vertebrae 1–12 as upper and lower limits for the spinal canal. For each of the OARs, we propagated the contours from each of the respiratory phases to the AIP CT scan. Here, an IRV structure was generated.

We aimed to characterize patients with a clinically relevant increase in NTCPs when applying the IRV concept (retrospectively, without new optimization). First, we identified patients with a relevant increase in D1%, D2%, or maximum dose (Dmax) when comparing the dose for the OARs and the corresponding IRVs on the AIP CT scan (an increase of >5 Gy (bronchial tree, esophagus, trachea) or 0.5 Gy (spinal canal)). Please see Supplementary Figure S1 for a graphical presentation of the dose differences between the OARs and IRVs. In the patients with a relevant increase in these dose parameters, we calculated the NTCPs for the OARs and corresponding IRVs. For the spinal canal and the esophagus, we used the software, ‘RADBIOMOD’ (v.0.3b), with the Lyman–Kutcher–Burman model [22]. Since there is only limited data on NTCP calculations in the trachea and bronchial tree, we estimated the complication risks based on the models by Dujim et al. [23]. For these OARs, we calculated the maximum equivalent dose of 2 Gy per fraction (EQD_2_, α/β ratio = 3) and estimated the risks based on the NTCP models for any grade of radiographic toxicity in the lobar bronchi ([23], page 128). In previous studies, the risks (here, for proximal bronchial tree toxicity) were estimated in a similar way [24].

In the analyses on motion management, we compared volumes between the OARs on the AIP CT scan and the IRVs on the AIP CT scans. Additionally, we analyzed the deviation of the geometric centers of the OAR and the IRV structures. Therefore, the coordinates of the centers (x, y, z) were registered for the structures in each of the respiratory phases and the OARs in the AIP CT scan. We calculated the difference between the centers in each of the phases and the center of the IRV structure in the AIP CT scan. For further analysis, we considered the maximum difference for each structure. We tested for an influence of patient-related parameters (e.g., body height) on the volume differences and the distances between the geometric centers. Here, we used the Mann–Whitney U test and Spearman’s rank correlation (SPSS v. 27, IBM, Armonk, NY, USA). *p*-values < 0.05 were considered statistically significant.

## 3. Results

### 3.1. Clinical/SBRT Characteristics and Outcomes in Centrally vs. Peripherally Located Tumors

We compared characteristics and outcomes in patients with central and peripheral tumors (cut-off, distance ≤2 cm vs. >2 cm from central OAR (central airways, spinal canal, esophagus)). We found that the applied biologically effective dose (BED, alpha/beta ratio of 10 Gy) was significantly higher in patients with peripheral vs. central tumors (median 115.5 vs. 105 Gy, *p* = 0.001). In patients with central vs. peripheral tumors, a BED < 100 Gy was applied more frequently (27.3% vs. 5.4% of the patients, *p* = 0.006). The PTV volume was significantly higher in patients with central tumors (*p* = 0.046). The doses to lungs–GTV (i.e., the volume created by subtraction of the GTV from both lungs, differences in mean dose, V5Gy, and V20Gy) were higher in patients with central tumors (each, *p* < 0.05). SBRT application was incomplete in two patients (one patient with a central tumor, 7.5/60 Gy; one patient with a peripheral tumor, 52.5/60 Gy). Please see Table 1.

In the whole patient cohort (*n* = 78 patients), the 2-year overall survival rate was 56.8%. There were four patients with local progression, five patients with regional progression, and ten patients with distant progression. The median follow-up was 18.5 months (range, 0.6–65.5 months). At the end of follow-up, 36/78 patients (46.2%) were alive. Death was documented in 42/78 patients (53.8%). The causes of death were unknown in most of the patients (35/42 patients (83.3%)). In 7/42 patients (16.7%), the causes of death were tumor progression (*n* = 2), pneumonitis (*n* = 1), lung infection (*n* = 1), exacerbation of COPD (*n* = 1), cerebral mass with bleeding (potentially metastasis or vascular/ischemic cause, *n* = 1), and pancreatitis (*n* = 1).

**Figure 2:**
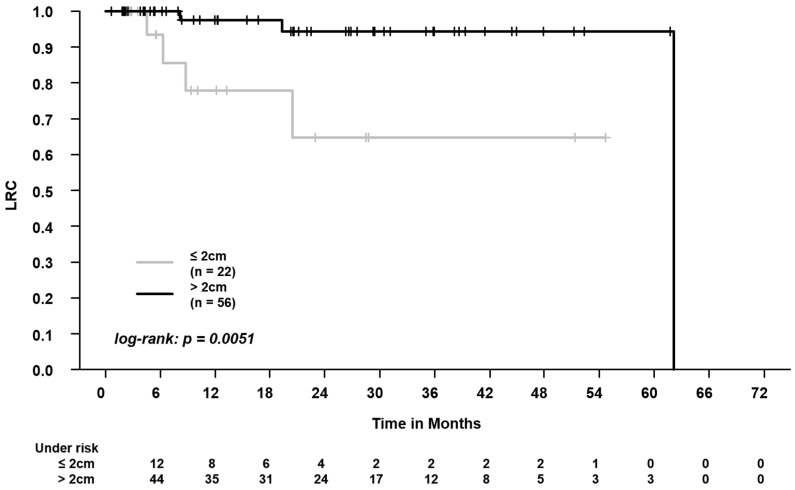
Comparison of locoregional control (LRC) in patients with centrally vs. peripherally located tumors (distance to organs at risk: central airways, spinal canal, esophagus, ≤2 cm vs. >2 cm). The 2-year LRC was 94.4% vs. 64.8%.

**Figure 3:**
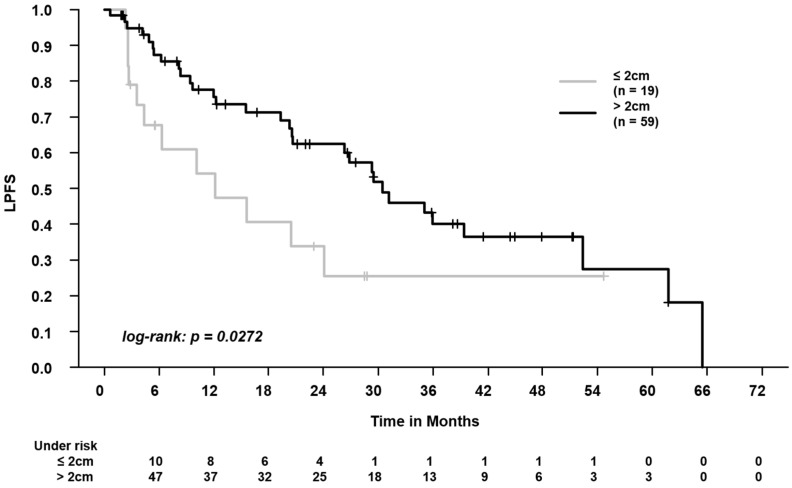
Comparison of locoregional progression-free survival (LPFS) in patients with tumors at a distance ≤2 cm vs. >2 cm from the central airways. The 2-year LPFS was 62.4% vs. 33.8%.

When comparing patients with central vs. peripheral tumors, we found worse LRC (2-year LRC: 64.8% vs. 94.4%, log-rank, *p* = 0.0051, Figure 2). When analyzing the differences in outcomes separately for the OARs, we found worse outcomes for central airways (PFS, LPFS (Figure 3), and LRC) and for the esophagus (OS, PFS; LPFS, and LRC), but not for the spinal canal (Please see Supplementary Table S1 for the results of the respective survival analyses).

### 3.2. Structure Movement Amplitudes and Volumes

For analyses on motion management, 35 patients with central tumors were studied. Herein, we present the mean values for the patient cohort. The maximum differences in geometric centers between the OARs in each respiratory phase and the IRVs on the AIP CT scan were 5.2 mm (bronchial tree), 4.2 mm (trachea), 5.5 mm (esophagus), and 4.3 mm (spinal canal). The absolute volume differences between OARs and IRVs were 22.0 cm^3^ (bronchial tree), 7.8 cm^3^ (trachea), 19.6 cm^3^ (esophagus), and 8.2 cm^3^ (spinal canal). Please see Table 2 for further details.

### 3.3. Influence of Clinical Characteristics on Structure Movement and Volume Changes

**Figure 4:**
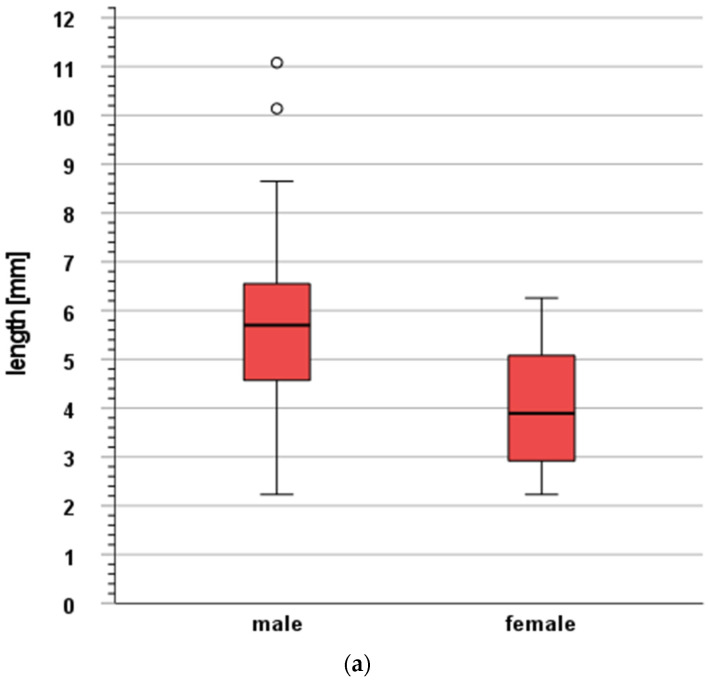

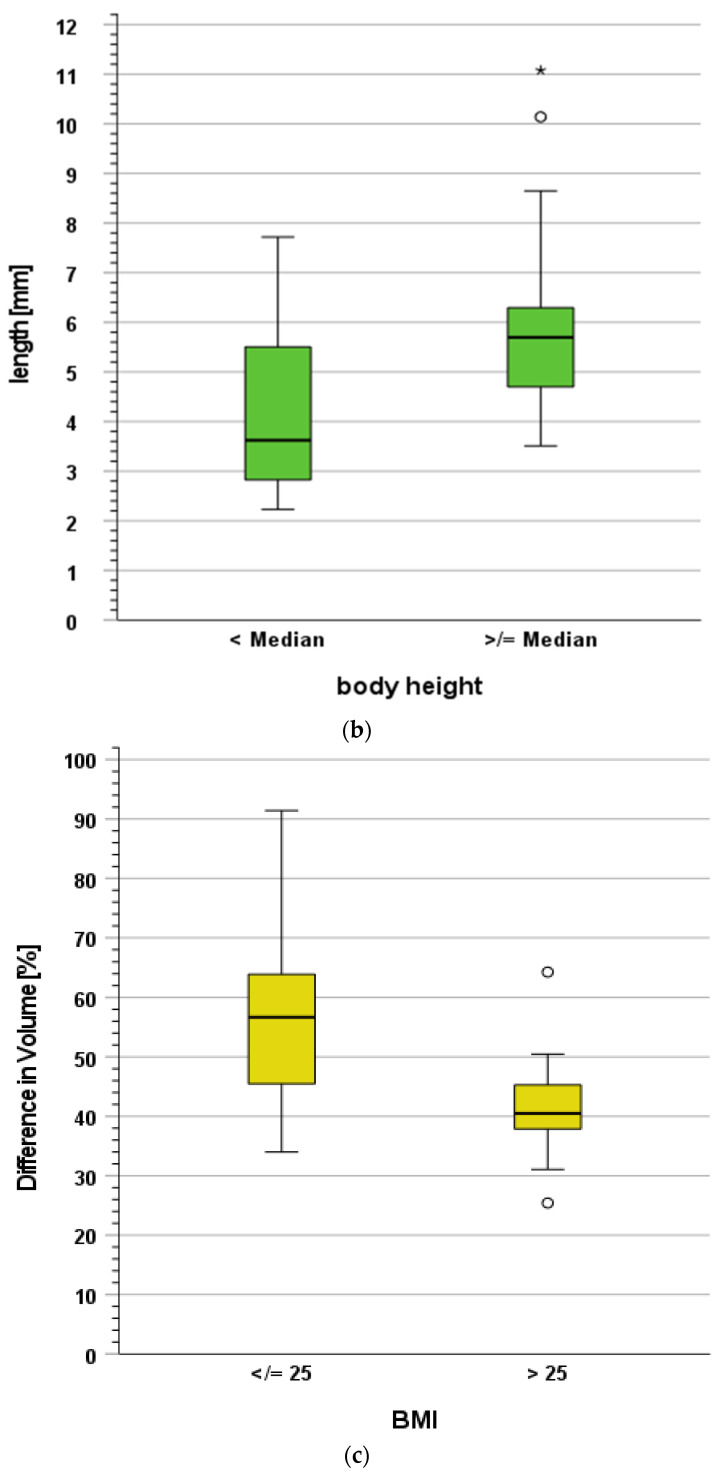
(**a**) Influence of gender on the maximum vector of movement for the bronchial tree (length, mm). Male patients had greater movement (median 5.7 mm vs. 3.9 mm, *p* < 0.05, 21 male patients vs. 14 female patients). (**b**) Influence of body height on the maximum vector of movement for the bronchial tree (length, mm). Tall patients had greater movement (median 5.7 mm vs. 3.6 mm, *p* < 0.05; cut-off median of body height [1.68 m], 16 patients with smaller height vs. 19 patients with height ≥ median). * Values that are more than 3x interquartile range below first quartile or above third quartile. (**c**) Influence of the body mass index (BMI) on the volume changes of the esophagus. Here, we compared organs at risk and corresponding internal organs at risk volumes. Patients with high BMI had lower volume changes (median 56.7% vs. 40.5%, *p* < 0.05, cut-off 25 kg/m^2^, 13 patients with BMI ≤ 25 kg/m^2^ vs. 22 patients with higher BMI).

Furthermore, we tested for an influence of clinical characteristics on structure movement and volume changes in the 35 patients with central tumors. Here, we considered the patient’s age, gender, body height, weight, and body mass index (BMI). In Figure 4a–c, we present the parameters with a significant influence on movement or volume changes. We found a greater maximum vector of movement for the bronchial tree in males (Figure 4a) and in tall patients (Figure 4b, cut-off, median of 1.68 m). Additionally, we found fewer volume changes for the esophagus in patients with high body mass index (BMI, >25 kg/m^2^, Figure 4c). Please see Supplementary Table S2 for a detailed analysis of the influence of all the clinical characteristics.

### 3.4. Influence of the Internal Organ at Risk Volume (IRV) Concept on Normal Tissue Complication Probabilities (NTCPs)

**Figure 5:**
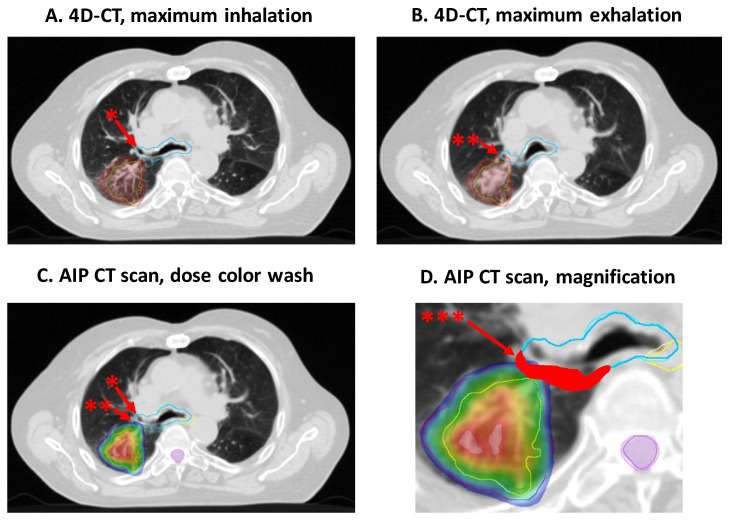
Illustration of the respiration-dependent movement of the bronchial tree. Patient with a stage IIA adenocarcinoma of the right upper lobe. SBRT was applied up to 60 Gy in 8 fractions prescribed on the 80% isodose using VMAT. The images depict the corresponding slices of the 4D-CT scan with maximum inhalation (**A**) and maximum exhalation (**B**). In (**C**) (average intensity projection CT scan), the dose is shown from 60 Gy (blue) to 75 Gy (red) with the contours of the bronchial tree in turquoise (* maximum inhalation, ** maximum exhalation). In the image (**D**) and magnification of image (**C**), the gain in volume between internal organ at risk volume (IRV) and organ at risk (OAR) is marked (***). The distance between the GTV and the bronchial tree was 3 mm. Please note the relevant volume difference between OAR and IRV in proximity to the target volume. The absolute difference in volume between the bronchial tree (59.0 cm^3^) and its corresponding IRV (94.4 cm^3^) was 35.5 cm^3^ (relative increase in volume, 60%). The maximum difference in geometric centers between OAR and IRV was 10.1 mm. The absolute difference in maximum dose was 8.4 Gy (OAR, 62.9 Gy vs. IRV, 71.2 Gy). The absolute increase in normal tissue complication probability, when comparing OAR vs. IRV, was 11.5% (relative increase, 44.2%).

Here, we aimed to characterize patients with a clinically relevant increase in NTCPs among the 35 patients with central tumors when applying the IRV concept (retrospectively, without new optimization). First, we identified 12 patients with a relevant increase in dosimetric parameters when comparing OARs and IRVs (please see Section 2.3.2 and Supplementary Figure S1). In these patients, the NTCPs for OARs and corresponding IRVs were calculated (Supplementary Table S3, differences in maximum doses and NTCPs for these patients). The mean absolute increase in NTCPs was 5.5% (0–22.5%), and the mean relative increase was 54.78% (0–181%). Please see Table 3 and Supplementary Table S3. Please see Figure 5 for an illustration of the respiration-dependent movement of the bronchial tree and its influence on NTCPs.

## 4. Discussion

SBRT yields excellent local tumor control in patients with early-stage NSCLC when indicated, e.g., in elderly patients or in patients who refuse surgery [2]. At the same time, treatment can be associated with relevant complications, especially in patients with a central tumor location [10,25]. Previous authors hypothesized that the PRV/IRV concept might improve toxicity profiles [13,14,15]. Nardone et al. applied the IRV concept in patients with SBRT for NSCLC. When taking the organ motion into account, 42% of the radiotherapy plans were unacceptable [15]. This work aims at (1) comparing clinical aspects in central vs. peripheral tumors, (2) applying the IRV concept in central tumors (retrospectively, without new optimization), (3) analyzing organ motion, and (4) studying associated NTCPs.

We found that, in patients with central tumors, the median BED was significantly lower (central, median 105 Gy vs. peripheral, median 115.5 Gy). A BED of <100 Gy was applied in 27.3% of patients with central tumors vs. 5.4% of patients with peripheral tumors. Patients with central tumors had higher PTV volumes and higher doses to lungs–GTV (mean dose, V5Gy, and V20Gy). Furthermore, we found worse LRC in patients with central tumors (2-year LRC of 64.8% vs. 94.4% in patients with peripheral tumors). When analyzing the outcomes separately for the OAR, we found worse outcomes for the central airways and for the esophagus, but not for the spinal canal.

Our results are in line with previous studies on comparisons of central vs. peripheral tumors in SBRT of early-stage NSCLC. These studies found lower BED (mean 120.2 vs. 143.5 Gy [5]), larger tumor size (as we found larger PTV volumes, with tumors of mean 1.9 vs. 2.5 cm and median 2.6 vs. 3.1 cm in peripheral vs. central location [5,26]), higher lung doses (V5Gy, V20Gy [27]), and worse local control (freedom from local progression, 52% (central) vs. 84% (peripheral) [26]) in central tumors. Additionally, previous studies demonstrated that a BED_10Gy_ of ≥100 Gy is associated with higher local control, and that dose escalation increases local control/overall survival and complications [28]. In the presented study, in line with these findings, patients with central lesions received a BED_10Gy_ of <100 Gy in a higher percentage and, consecutively, experienced worse LRC.

In conclusion, in clinical routine, the perception of increased complication risks leads to insufficient doses and reduced outcomes in patients with centrally located tumors [26]. In spite of the perception of high complication risks, previous studies found lower rates of ≥grade 3 acute toxicities [5] and low overall rates of toxicities [26] in patients with central tumors. Thus, in clinical routine, balancing dose escalation/tumor control and complication risks (especially concerning the central airways [25]) is very challenging and controversially discussed [29]. In this context, motion management strategies are very important [11]. When active motion management techniques are not available/applicable, safety margins/4D-CT and internal motion have to be considered [11]. However, data on the internal motion of central organs at risk in SBRT of early-stage NSCLC are very rare (e.g., [15]).

When applying the IRV concept (retrospectively, without new optimization), we found maximum differences in the geometric centers of mean 4.2 mm (trachea), 4.3 mm (spinal canal), 5.2 mm (bronchial tree), and 5.5 mm (esophagus). The relative differences in volume (IRV–OAR, mean) were 16.1% (spinal canal), 19.4% (trachea), 40.9% (bronchial tree), and 47.5% (esophagus). The maximum vector of movement for the bronchial tree was greater in males and in tall patients. The volume changes (IRV–OAR) in the esophagus were lower in patients with high BMI.

In the literature, specific data on thoracic organ motion are very rare. When considering our results and the study by Nardone et al. [15], there is evidence that there are relevant volume differences (IRVs–OARs). In detail, the authors reported a difference of 4% (spinal cord), 23% (trachea), and 25% (esophagus) (proximal bronchial tree, absolute difference of 18 vs. 26 cm^3^, relative difference not reported, thus, putatively, 44%) [15]. Zhang et al. used the IRV concept for the thoracic and abdominal organs. However, the discussed central OARs (bronchial tree, trachea, esophagus, and spinal canal) were not studied by Zhang et al. [16]. To the authors’ knowledge, a putative relationship between patient characteristics and structure movement/volume changes has not been reported. However, an analysis seems reasonable, since previous studies found evidence for increased risks of toxicity associated with SBRT of NSCLC for females (here, for pneumonitis [30]) or obese patients (here, for chest wall pain [31]). After all, our results indicate that organ motion could be more pronounced in males and tall patients, whereas it could be less pronounced in patients with higher BMI. This could have implications for risk-adapted strategies in motion management or toxicity monitoring in these patient groups.

Finally, when comparing OARs and IRVs, we found a relevant increase in dosimetric parameters (D1%, D2%, Dmax) in 12/35 patients (34.3%) with central tumors. Nardone et al. found unacceptable radiotherapy plans in 42% of the patients (here, in 63% of the patients, the tumors were located centrally) [15]. These findings demonstrate that the application of the IRV concept has a relevant impact on dosimetric parameters and radiotherapy plans. In further analysis of the 12 patients in our study, the mean absolute increase in NTCPs was 5.5% (0–22.5%), and the mean relative increase was 54.78% (0–181%). It can be assumed that an absolute increase of ≥10% in NTCPs could be clinically relevant. This was documented in three patients for the bronchial tree. In these cases, the distance between the tumor and the bronchial tree was 0.3–1.4 cm.

When considering relevant complications in SBRT for central tumors, larger prospective studies pointed towards a particular relevance of the bronchial tree (e.g., Lindberg et al., 8/10 cases of treatment-related death occurred due to bronchopulmonary hemorrhage [25]). The region of ≤2 cm around the proximal bronchial tree is generally considered the “no-fly zone”, with an increased risk of relevant toxicities [10,32]. Studies have made efforts to further subclassify this area, e.g., when analyzing tumors located nearer (≤1 cm) to the bronchial tree [33]. Recently, Lindberg et al. refined the risk factors for toxicity by considering the dose to further substructures (here, mainstem, intermediate, and lobar bronchi) [34]. However, when applying the IRV concept, our study found an increase of ≥10% in NTCPs in three patients with tumors 0.3–1.4 cm from the bronchial tree. Thus, when it comes to these distinct differences or very small substructures, motion management (as a possibility, using the IRV concept) should come into focus. However, as Noël et al. pointed out in 2022, considering the PRV concept in general—albeit already described in 2006—neither definition, purpose, nor dose constraints exist [14,35]. Studies on the IRV concept in thoracic OARs are very rare but could increase the perception of OAR motion in SBRT, in analogy to Galileo’s famous comment, ‘and yet it moves’ [15,16,36]. Finally, recent developments include the implementation of magnetic resonance imaging (MRI) in linear accelerators with the opportunity of real-time tracking for target volumes (four-dimensional MRI, 4D-MRI) [37]. There is evidence that 4D-MRI is a promising technique for lung tumor delineation and motion assessment with greater robustness against inter-fractional changes than 4D-CT-based radiotherapy [37]. Previous studies have reported results when applying 4D-MRI for target volumes [37], whereas thoracic OARs were studied less frequently [38]. Thus, when considering motion management in SBRT for central lung tumors, 4D-MRI can be considered a promising technology for an optimal balance of toxicities and tumor control [39].

## 5. Conclusions

The IRV concept might improve toxicity profiles in SBRT for NSCLC [13,14,15]. We studied (1) clinical aspects in central vs. peripheral tumors, (2) the IRV concept in central tumors, (3) organ motion, and (4) associated NTCPs. We found lower biologically effective doses, larger planning target volume sizes, higher lung doses, and worse locoregional control for central tumors. This emphasizes the need to optimize methods to balance dose escalation with toxicities in central tumors. Organ motion was greater in males and tall patients (both, for the bronchial tree), whereas volume changes were lower in patients with higher BMI (for the esophagus). This could have implications for risk-adapted strategies in motion management or toxicity monitoring in these patient groups. Applying the IRV concept (retrospectively, without new optimization), we found an absolute increase of >10% in NTCPs for the bronchial tree in three patients (distance from the bronchial tree, 0.3–1.4 cm). Recent studies made efforts to further subclassify central tumors in the “no-fly zone”, either by exact distance or by substructures (e.g., mainstem, intermediate, and lobar bronchi) [33,34]. Based on the NTCP increase in our study, when it comes to these distinct differences or very small sub-structures, motion management (as a possibility, using the IRV concept) should come into focus.

## Figures and Tables

**Table 1:** Comparison of clinical/stereotactic body radiation therapy (SBRT) characteristics in patients with centrally vs. peripherally located tumors (distance to OARs (organs at risk): central airways, spinal canal, esophagus, ≤2 cm vs. >2 cm). ECOG: Eastern Cooperative Oncology Group status. UICC: Union Internationale Contre le Cancer, 8th edition. VMAT: volumetric modulated arc therapy. IMRT: intensity-modulated radiotherapy. 3DCRT: 3D conformal radiotherapy. GTV: gross tumor volume. The numbers (% of the patients) are given if not otherwise specified. ^1^ In one patient, SBRT was applied simultaneously to a central tumor (44 Gy in 8 fractions) and a peripheral tumor (55 Gy in 5 fractions), as described in our previous study [18]. Here, we decided to include this patient in the group with central tumors. The stages were cT1a and cT1b (counted as UICC stage I in the table). The radiotherapy doses (planned/applied/fractions) and GTV/PTV volumes of the central tumor are included in the table and were used for analysis. The technique was VMAT for both tumors. The dose summation for radiotherapy of both tumors was used for lungs–GTV. ^2^ Since this part of the study mainly focused on clinical outcomes, not on technical analysis of SBRT, we decided to include 3 patients with radiotherapy in 18 fractions [18]. As mentioned in our previous study, formally, the recent literature defines SBRT as radiotherapy in a maximum of 12 fractions [11,18]. ^3^ Pneumonitis: 9 patients with grade 1, 6 patients with grade 2, and 2 patients with grade 3. ^4^ Pearson’s Chi-squared test. ^5^ Mann–Whitney U test.

Parameter	Central Tumors (*n* = 22 Patients)	Peripheral Tumors (*n* = 56 Patients)	*p*-Value
Gender			0.32 ^4^
Male	16 (72.7)	34 (60.7)	
Female	6 (27.3)	22 (39.3)	
ECOG (median, min, max)	1 (0–2)	1 (0–4)	0.3 ^5^
UICC stages ^1^			0.96 ^4^
I	17 (77.3)	43 (76.8)	
II–III	5 (22.7)	13 (23.2)	
Planned dose ^1^ [Gy, median, min–max)]	60 (44–60)	55 (54–60)	0.28 ^5^
Planned fractions ^1,2^ (median, min–max)	8 (3–18)	5 (3–18)	0.002 ^5^
Planned dose ^1^ [biologically effective dose, α/β = 10 Gy, Gy, median, min–max]	105 (68.2–151.2)	115.5 (70.2–151.2)	0.001 ^5^
Applied dose ^1^ [biologically effective dose, α/β = 10 Gy, Gy, median, min–max]	105 (13.13–151.2)	115.5 (70.2–151.2)	0.001 ^5^
Applied dose < 100 Gy ^1^ [biologically effective dose, α/β = 10 Gy]	6 (27.3)	3 (5.4)	0.006 ^4^
Radiotherapy technique ^1^			0.83 ^4^
VMAT/IMRT	20 (90.9)	50 (89.3)	
3DCRT	2 (9.1)	6 (10.7)	
GTV volume ^1^ [cm^3^, median, min–max]	32.55 (2.7–141.9)	14.8 (1.4–119.4)	0.17 ^5^
PTV volume ^1^ [cm^3^, median, min–max]	90.26 (11.9–244.9)	45.0 (12.8–329.8)	0.046 ^5^
Lungs–GTV ^1^, Dmean [Gy]	5.8 (1.24–11.3)	3.7 (1.7–10.5)	0.009 ^5^
Lungs–GTV ^1^, V5Gy [%]	26.9 (4.9–56.2)	17.3 (5.9–49.9)	0.003 ^5^
Lungs–GTV ^1^, V20Gy [%]	8.0 (0–15.6)	5.5 (2.0–18.7)	0.02 ^5^
Pneumonitis ^3^	5 (22.7)	12 (21.4)	0.90 ^4^

**Table 2:** Structure movement amplitudes and volume differences. Comparison of organs at risk (OARs) volumes in each respiratory phase scan and internal organs at risk volumes (IRVs) on average intensity projection (AIP) CT scan. The mean (min–max) values are presented.

Parameter	Bronchial Tree	Trachea	Esophagus	Spinal Canal
Maximum difference in geometric centers, OAR (each respiratory phase), and IRV on AIP CT scan [mm]	5.2 (2.2–11.1)	4.2 (1.4–14.6)	5.5 (2.0–13.2)	4.3 (1.5–9.7)
OAR volumes on AIP CT scan [cm^3^]	54.4 (34.7–85.4)	40.5 (22.3–57.2)	42.0 (25.7–74.2)	57.2 (33.5–90.0)
IRV volumes on AIP CT scan [cm^3^]	76.4 (46.8–116.1)	48.3 (27.2–75.7)	61.5 (37.4–103.9)	65.4 (48.9–88.7)
Absolute difference, IRV–OAR volume [cm^3^]	22.0 (10.5–35.5)	7.8 (2.7–18.8)	19.6 (11.4–33.7)	8.2 (−1.3–20.2)
Relative difference, IRV–OAR volume [%]	40.9 (26.9–60.4)	19.4 (6.6–53.5)	47.5 (25.4–91.4)	16.1 (−1.4–57.4)

**Table 3:** Differences in normal tissue complication probabilities (NTCPs) when comparing organs at risk (OARs) and internal organs at risk volumes (IRVs). Please see Section 2.3.2, Supplementary Figure S1 and Supplementary Table S3 for further details. The NTCPs for the bronchial tree and the trachea were estimated using the graphs for the maximum dose (in EQD2) for the bronchial tree by Dujim et al. [23]. The NTCPs for the esophagus and the spinal canal were calculated using the software, ‘RADBIOMOD’, with the Lyman–Kutcher–Burman model ([22]; please see Section 2.3.2 for further details).

Structure	Patient No.	Distance between Tumor and Structure [cm]	NTCP of OAR [%]	NTCP of IRV [%]	NTCP, Absolute Increase [%]	NTCP, Relative Increase [%]
Bronchial Tree	1	1.4	17.0	34.0	17.0	100.0
	2	0.0	63.0	66.0	3.0	4.8
	3	0.3	26.0	37.5	11.5	44.2
	4	0.0	23.0	32.0	9.0	39.1
	5	0.0	45.0	48.0	3.0	6.7
	6	1.1	35.5	58.0	22.5	63.4
Esophagus	3	2.4	2.1	5.9	3.8	181.0
	7	0.3	1.5	4.0	2.5	166.7
Spinal Canal	1	2.2	0.0	0.0	0.0	0.0
	8	1.8	<0.01	<0.01	0.0	0.0
	9	4.1	<0.01	<0.01	0.0	0.0
Trachea	6	4.1	4.0	5.0	1.0	25.0
	10	1.2	4.0	10.5	6.5	162.5
	11	3.5	4.5	5.5	1.0	22.2
	12	1.0	24.5	26.0	1.5	6.1

## Data Availability

The data presented in this study are available on reasonable request from the corresponding author.

## Supplementary Materials

The following supporting information can be downloaded at: https://www.mdpi.com/article/10.3390/cancers16010231/s1, Figure S1: Absolute dose differences [Gy] between organ at risk and internal organs at risk volumes. The dose differences are shown as a function of the distance of the tumors to the respective structure. Each dot (green, D1%, yellow, D2%, red, Dmax) represents the dose difference for one patient. The dashed line marks the cut-off for a relevant dose increase (please see Section 2.3.2). Table S1: Cox regression analysis, distance of the tumor to organs at risk, and outcomes. HR: hazard ratio. OS: overall survival. PFS: progression-free survival. LPFS: local progression-free survival. LRC: locoregional control. CI: confidence interval. ^1^ Central organs at risk (OARs): central airway, esophagus, and spinal canal. Table S2: Influence of clinical characteristics on structure movement and volume changes. The Mann–Whitney U test was used to test for an influence of the parameters. P-values are given for each parameter. SBRT: stereotactic body radiation therapy. OAR: organ at risk. IRV: internal organ at risk volumes. AIP: average intensity projection. Table S3: Differences in maximum doses and normal tissue complication probabilities (NTCPs) when comparing organ at risk (OAR) and internal organ at risk volumes (IRV). Patients with relevant increases in dosimetric parameters were preselected (Section 2.3.2, Supplementary Figure S1). For these patients (*n* = 12), we present the distance of the tumor to the structures, the maximum doses, and the NTCPs. The NTCPs for the bronchial tree and the trachea were estimated using the graphs for the maximum dose (in EQD2) for the bronchial tree by Dujim et al. The NTCPs for the esophagus and the spinal canal were calculated using the software, ‘RADBIOMOD’, with the Lyman–Kutcher–Burman model (please see Section 2.3.2 for further details).
