# Supplementary material for: And Yet It Moves: Clinical Outcomes and Motion Management in Stereotactic Body Radiation Therapy (SBRT) of Centrally Located Non-Small Cell Lung Cancer (NSCLC): Shedding Light on the Internal Organ at Risk Volume (IRV) Concept
Source: Cancers (Basel). 2024 Jan 4;16(1):231. doi: 10.3390/cancers16010231 (PMC10778176; doi:10.3390/cancers16010231)
Supplement: Supplementary file 1 [file cancers-16-00231-s001.zip › Suppl. Table S1.pdf]

Suppl. Table S1. Cox regression analysis, distance of the tumor to organs at risk and outcomes. HR: hazard ratio. OS: overall survival. PFS: progression-free survival. LPFS: local progression-free survival. LRC: locoregional control. CI: confidence interval. [1]Central organs at risk (OARs): central airway, esophagus, or spinal canal.

	OS		PFS		LPFS		LRC	
Distance of the tumor to the structures (cutoff, [numbers of patients])	HR (95% CI)	*p* value	HR (95% CI)	*p* value	HR (95% CI)	*p* value	HR (95% CI)	*p* value
Central airway (≤2cm [19] vs. >2cm, [59])	0.58 (0.29-1.17)	0.129	0.46 (0.23-0.9)	0.024	0.43 (0.22-0.86)	0.017	0.09 (0.02-0.51)	0.006
Esophagus (≤2cm [9] vs. >2cm, [69])	0.32 (0.14-0.76)	0.009	0.26 (0.11-0.6)	0.002	0.22 (0.09-0.52)	0.001	0.05 (0.01-0.28)	<0.001
Spinal canal (≤2cm [4] vs. >2cm, [75])	1.01 (0.24-4.19)	0.994	1.01 (0.24-4.2)	0.987	1.14 (0.28-4.76)	0.853	22.34 (<0.01-8.1*10^7)	0.687
Central OARs[1] (≤2cm [22] vs. >2cm, [56])	0.67 (0.34-1.33)	0.250	0.56 (0.29-1.09)	0.087	0.53 (0.27-1.04)	0.064	0.13 (0.02-0.7)	0.018
