# Supplementary material for: And Yet It Moves: Clinical Outcomes and Motion Management in Stereotactic Body Radiation Therapy (SBRT) of Centrally Located Non-Small Cell Lung Cancer (NSCLC): Shedding Light on the Internal Organ at Risk Volume (IRV) Concept
Source: Cancers (Basel). 2024 Jan 4;16(1):231. doi: 10.3390/cancers16010231 (PMC10778176; doi:10.3390/cancers16010231)
Supplement: Supplementary file 1 [file cancers-16-00231-s001.zip › Suppl. Table S2.pdf]

Suppl. Table S2. Influence of clinical characteristics on structure movement and volume changes. The Mann-Whitney-U test was used to test for an influence of the parameters. P-values are given for each parameter. SBRT: stereotactic body radiation therapy. OAR: organs at risk. IRV: internal organ at risk volumes. AIP: average-intensity projection.

	Bronchial tree		Trachea		Esophagus		Spinal canal	
	Maximum difference in geometric centers, OAR (each respiratory phases) and IRV on AIP CT scan [mm]	Relative difference, IRV-OAR volume [%]	Maximum difference in geometric centers, OAR (each respiratory phases) and IRV on AIP CT scan [mm]	Relative difference, IRV-OAR volume [%]	Maximum difference in geometric centers, OAR (each respiratory phases) and IRV on AIP CT scan [mm]	Relative difference, IRV-OAR volume [%]	Maximum difference in geometric centers, OAR (each respiratory phases) and IRV on AIP CT scan [mm]	Relative difference, IRV-OAR volume [%]
Age [years], cut-off: median (72.4, range 57.2-89.8)	0.438	0.621	0.210	0.355	0.291	0.092	0.908	0.113
Gender, male (n=21) vs. female (n=14)	**0.007**	0.602	0.480	0.201	0.522	0.501	0.419	0.613
Body heigth [m], cut-off: median (1.68, range 1.48-1.84)	**0.005**	0.540	0.947	0.389	0.529	0.573	0.417	0.741
Weight [kg], cut-off: median (75.0, range 49.0-110)	0.777	0.739	0.868	1.000	0.790	**0.014**	0.182	0.405
Body Mass Index [kg/m^s], cut-off: median (26,7, range 19.1-38.3)	0.754	1.000	0.373	0.817	0.817	**0.012**	0.843	0.373
Body Mass Index [kg/m^s], cut-off: >25kg/m^2	0.720	0.838	0.946	0.562	0.946	**0.002**	0.946	0.973
