# Supplementary material for: And Yet It Moves: Clinical Outcomes and Motion Management in Stereotactic Body Radiation Therapy (SBRT) of Centrally Located Non-Small Cell Lung Cancer (NSCLC): Shedding Light on the Internal Organ at Risk Volume (IRV) Concept
Source: Cancers (Basel). 2024 Jan 4;16(1):231. doi: 10.3390/cancers16010231 (PMC10778176; doi:10.3390/cancers16010231)
Supplement: Supplementary file 1 [file cancers-16-00231-s001.zip › Suppl. Table S3.pdf]

Suppl. Table S3. Differences in maximum doses and normal tissue complication probabilities (NTCPs) when comparing organ at risk (OAR) and internal organ-at-risk volume (IRV). Patients with relevant increase in dosimetric parameters were preselected (section 2.3.2, Suppl. Figure S1). For these patients (n=12), we present the distance of the tumor to the structures, the maximum doses, and the NTCPs. The NTCPs for the bronchial tree and the trachea were estimated using the graphs for the maximum dose (in EQD2) for the bronchial tree by Dujim et al. The NTCPs for the esophagus and the spinal canal were calculated using the the software 'RADBIOMOD' with the Lyman-Kutcher-Burman model (please see section 2.3.2 for further details).

Structure	Patient no.	Distance between tumor and structure [cm]	Dmax of OAR [Gy]	Dmax of IRV [Gy]	ΔDmax [IRV-OAR, Gy]	Dmax, EQD2 of OAR [Gy]	NTCP of OAR [%]	Dmax, EQD2 of IRV [Gy]	NTCP of IRV [%]
Bronchial Tree	1	1.4	53.24	68.70	15.46	102.81	17.0	159.22	34.0
	2	0.0	69.55	70.89	1.33	235.22	63.0	243.55	66.0
	3	0.3	62.86	71.23	8.37	136.51	26.0	169.58	37.5
	4	0.0	49.04	55.25	0.18	125.62	23.0	155.25	32.0
	5	0.0	75.81	77.69	0.77	189.16	45.0	197.51	48.0
	6	1.1	45.59	53.41	6.21	165.92	35.5	222.22	58.0
Esophagus	3	2.4	34.64	45.38	10.74	94.00	2.1	121.90	5.9
	7	0.3	30.70	46.63	15.93	85.20	1.5	125.50	4.0
Spinal Canal	1	2.2	23.90	24.79	0.89	71.80	0.0	73.40	0.0
	8	1.8	18.12	19.29	1.17	62.30	< 0.01	64.10	< 0.01
	9	4.1	18.72	19.85	1.13	63.20	< 0.01	65.00	< 0.01
Trachea	6	4.1	9.19	14.74	5.55	8.89	4.0	17.53	5.0
	10	1.2	10.90	55.87	44.97	7.86	4.0	68.19	10.5
	11	3.5	10.68	21.17	10.49	9.26	4.5	23.91	5.5
	12	1.0	61.40	62.98	1.57	131.09	24.5	136.95	26.0
