# Supplementary figures and images for: And Yet It Moves: Clinical Outcomes and Motion Management in Stereotactic Body Radiation Therapy (SBRT) of Centrally Located Non-Small Cell Lung Cancer (NSCLC): Shedding Light on the Internal Organ at Risk Volume (IRV) Concept
Source: Cancers (Basel). 2024 Jan 4;16(1):231. doi: 10.3390/cancers16010231 (PMC10778176; doi:10.3390/cancers16010231)

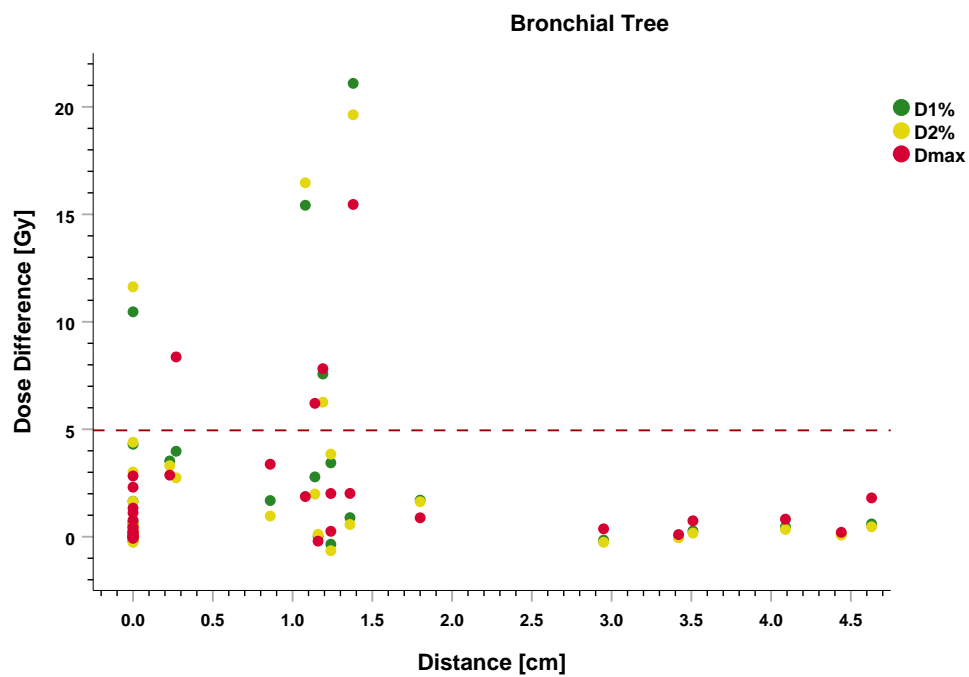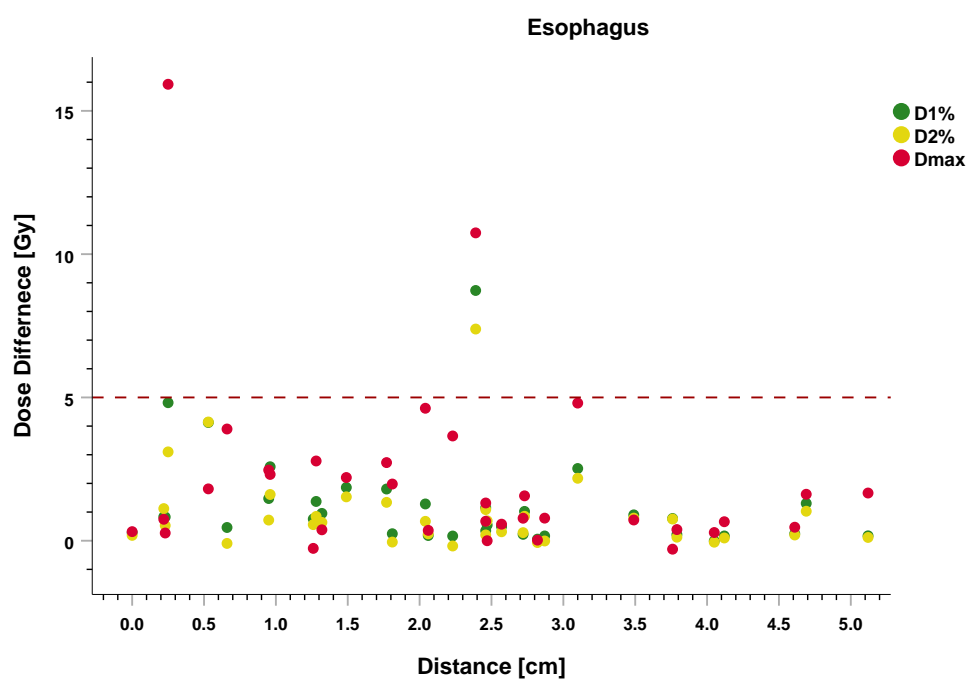

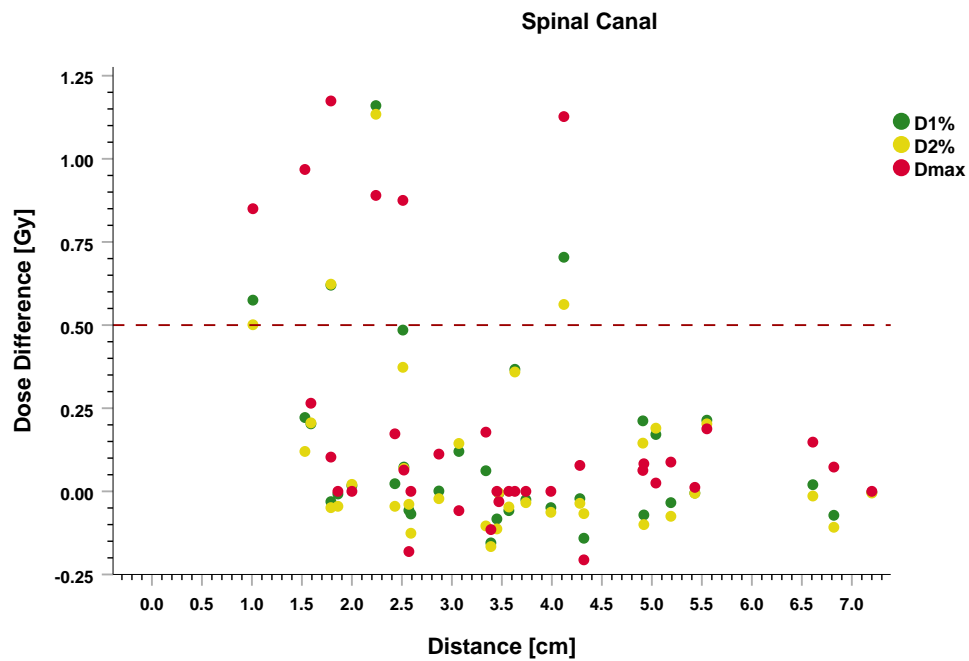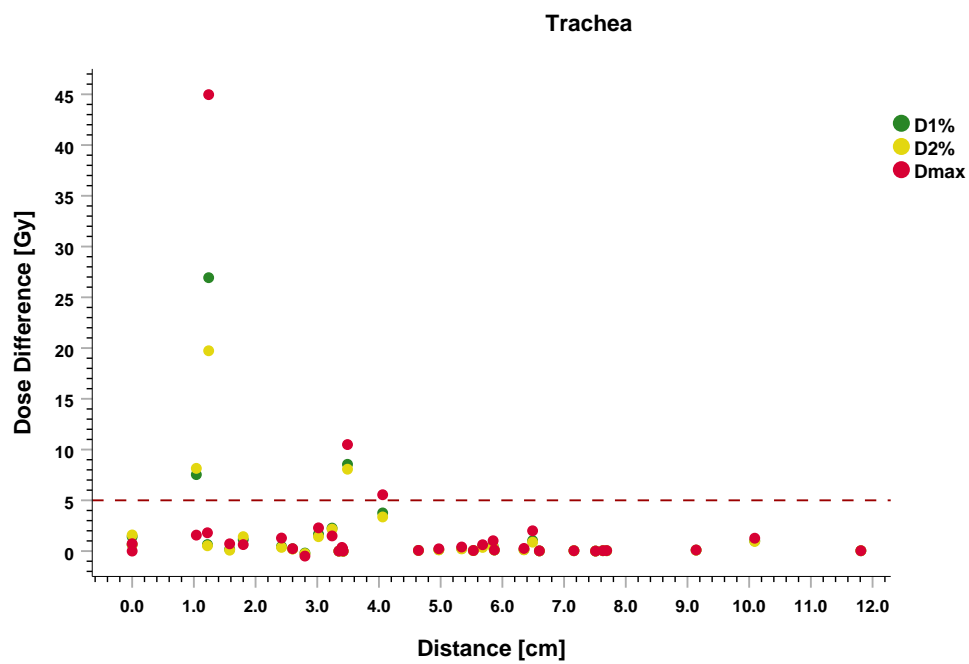

Supplement: Supplementary file 1 [file cancers-16-00231-s001.zip › Suppl. Figure S1.pdf]
